# ZnO Nanorod/Graphene Hybrid-Structures Formed on Cu Sheet by Self-Catalyzed Vapor-Phase Transport Synthesis

**DOI:** 10.3390/nano11020450

**Published:** 2021-02-10

**Authors:** Hak Dong Cho, Deuk Young Kim, Jong-Kwon Lee

**Affiliations:** 1 Quantum Functional Semiconductor Research Center, Dongguk University, Seoul 04620, Korea; hakcho1@dongguk.edu; 2 Division of Physics and Semiconductor Science, Dongguk University, Seoul 04620, Korea; 3 Division of Energy and Optical Technology Convergence, Cheongju University, Cheongju-si, Chungcheongbuk-do 28503, Korea

**Keywords:** ZnO nanorod, graphene, Cu sheet, self-catalyzed synthesis, hybrid-nanojunction, UV device

## Abstract

High crystalline ZnO nanorods (NRs) on Zn pre-deposited graphene/Cu sheet without graphene transfer process have been fabricated by self-catalyzed vapor-phase transport synthesis. Here, the pre-deposited Zn metal on graphene not only serves as a seed to grow the ZnO NRs, but also passivates the graphene underneath. The temperature-dependent photoluminescence spectra of the fabricated ZnO NRs reveal a dominant peak of 3.88 eV at 10 K associated with the neutral-donor bound exciton, while the redshifted peak by bandgap shrinkage with temperature and electron-lattice interactions leads a strong emission at 382 nm at room temperature. The optical absorption of the ZnO NRs/graphene hetero-nanostructure at this ultraviolet (UV) emission is then theoretically analyzed to quantify the absorption amount depending on the ZnO NR distribution. By simply covering the ZnO NR/graphene/Cu structure with the graphene/glass as a top electrode, it is observed that the current-voltage characteristic of the ZnO NR/graphene hetero-nanojunction device exhibits a photocurrent of 1.03 mA at 3 V under a light illumination of 100 μW/cm^2^. In particular, the suggested graphene/ZnO NRs/graphene hybrid-nanostructure-based devices reveal comparable photocurrents at a bidirectional bias, which can be a promising platform to integrate 1D and 2D nanomaterials without complex patterning process for UV device applications.

## 1. Introduction

Zinc oxide (ZnO) has been widely used in photodetector, solar cell, and display applications [1,2,3] due to a wide bandgap (~3.37 eV), a high mobility (~5000 cm^2^/Vs), and a large exciton binding energy (~60 meV). Especially, its self-organized growth as single-crystalline nanostructures leads to quantum confinement and small size effects, which reveals novel properties in optical and electrical aspects [4,5,6] relative to their thin-film counterpart. Thus, ZnO nanostructures including nanowires and nanotubes have been widely adopted on developing various functional devices such as nanowire field-effect transistors [7], photoconductors [8], biosensors [9], and piezoelectric nanogenerators [10]. 

Graphene, a single layer of carbon atoms, has also superb physical properties such as high electrical conductivity, ultrahigh mobility, and high transparency [11]. It can be used as a 2D platform onto which various 1D semiconducting nanomaterials can be grown. Especially, graphene placed on a dielectric surface can catalyze the growth of well-aligned nanostructures [12]. Thus, the integration of the graphene and the 1D semiconducting nanostructures can highly improve the optical and electrical properties for these hybrid-structures in comparison with the semiconducting nanostructures only [13]. 

Compared with the solution process that has been widely adopted for implementing graphene-ZnO hybrid-structures [14,15], the physical-vapor-deposition can produce high-quality ZnO nanorods (NRs) with a few defects by a low-cost and fast process [16,17]. In particular, graphene and graphene-metal hybrid-structures as buffer layers on a substrate can control the density and orientation of the ZnO nanostructures [13]. High density and high aspect-ratio ZnO NRs on graphene can be achieved by using a thermal-vapor-deposition technique [16,17]. Here, the orientation of the nanostructures in this thermal-vapor-deposition growth can be varied depending on the type of substrate and a metal catalyst [18]. However, the metal catalysts used to regulate the nanostructure nucleation sites can contaminate nanostructures [19]. Biroju, R.K. et al. reported the fabrication of different ZnO nanostructures on graphene and few-layer graphene by the graphene-assisted controlled growth with Au catalyst via a thermal-vapor-deposition method [12]. Here, the intrinsic defects such as oxygen vacancies, Zn and antisite oxygen induced by the catalyst are formed along the surfaces of the ZnO nanostructures during the fabrication process, leading to broad absorption at visible wavelengths [20,21,22]. Meanwhile, the catalyst-free growth of ZnO nanostructure on multilayer graphene by thermal evaporation in O_2_ ambient was reported. The broken C-C bonding of graphene by heating at high temperature of 600 °C above in O_2_ ambient results in etch-pit formation [23]. Then, the nucleation rates of Zn on the graphene are affected by the breaking rates of C-C bonds of graphene. Here, the green emission related to the specific defects such as O vacancies and Zn interstitials also causes the recombination of the green luminescence. 

In order to use the ZnO nanostructures in ultraviolet (UV) device applications, it is necessary to develop highly crystalline ZnO NRs without the defects induced by metal catalysts or the defects formed on graphene during the fabrication process to utilize its superb optoelectronic properties. It is known that carbon materials including amorphous carbon, graphite flakes, and photoresists can be used as preferential nucleation sites for growing ZnO nanostructures [24]. Here, ZnO nanostructures grown on these carbon materials are randomly oriented when there exist rough surfaces of these materials. Thus, flat and thin carbon-film has to be utilized in vacuum condition to grow uniform c-axial ZnO nanostructures at a high deposition temperature. In this regard, using thin and uniform carbon layer without any metal catalyst is preferred to grow high crystalline ZnO NRs with strong UV emission. Moreover, graphene transfer process to implement graphene-based devices makes the corresponding process complex and induces unwanted physical defects of graphene, which can also cause visible emission by the defect induced recombination. Therefore, using chemical-vapor-deposition (CVD) graphene on Cu sheet as a substrate without foreign metal catalyst can be a promising way to produce high crystalline ZnO NRs for this purpose. Moreover, the absorption of light into the ZnO NRs can be further improved since the light transmitted through the ZnO NRs/graphene hybrid-structure is reflected by the Cu substrate. 

In this respect, we firstly demonstrate the fabrication of vertically aligned ZnO NR on graphene/Cu sheet by using self-catalyzed vapor-phase transport (VPT) synthesis. The growth of ZnO NRs on Zn pre-deposited graphene/Cu sheet was performed at 700 °C using a thermal-vapor-deposition technique. The pre-deposited Zn-metal on graphene has a role of a seed to grow the ZnO NR as well as of passivating the graphene underneath. Then, the fabricated ZnO NRs on graphene/Cu sheet were investigated by the low temperature-dependent photoluminescence (PL) spectra ranging from 10 to 140 K. To elucidate the optical absorption depending on the ZnO NR distribution, we carried out the optical simulation for the emission peak (382 nm) determined by the PL measurement at room temperature. By simply covering the ZnO NRs/graphene/Cu sheet with the graphene/glass as an electrode, we fabricated hetero-nanojunction devices in a large area (~cm^2^ scale). Then, the fabricated graphene/ZnO NRs/graphene hybrid-nanojunction devices were characterized by measuring the current-voltage (I-V) curves under dark and illumination conditions.

## 2. Experimental Section

### 2.1. Graphene Synthesis on Cu Sheet

Graphene was synthesized on Cu sheet by the CVD process with a mixture of methane and hydrogen as described in detail elsewhere [25,26,27]. That is, the horizontal quartz tube (length: 2400 mm, diameter: 152 mm) was placed in the six-zone furnace (length: 1500 mm). A 25 μm-thick Cu foil (thickness: 25 μm, OFC, Alpa Aesar, 99.995%, Ward Hill, MA, USA) was loaded in the CVD chamber, which was then pumped to the base vacuum pressure (<10^−^^4^ Torr). Here, the Cu foil was vertically placed on a substrate holder in the central isothermal growth zone of the reactor during the growth process. The temperature of the chamber was ramped up to 1060 °C with a mixture of Ar (2000 sccm, 99.9999%) and H_2_ (30 sccm, 99.9999%) at the processing pressure of 470 mTorr. The Cu foil was typically annealed for 2 h. The furnace temperature was then decreased to the process temperature of 1020 °C with 30 sccm of H_2_ gas at 570 mTorr. When the furnace temperature reached 1020 °C, graphene layers started to grow on the Cu foil by the mixed gases of CH*_4_* (40 sccm, 99.9995%) and H_2_ (100 sccm, 99.9999%) while flowing 2000 sccm of Ar as a carrier gas to the reaction chamber at 600 mTorr for 30 min. The reaction chamber was then cooled down to room temperature with an average rate of 14 °C/min under the same flow rates of Ar and H_2_ gases without methane. 

### 2.2. Fabricaton of ZnO NRs on Graphene/Cu Sheet

After loading the graphene/Cu sheet in the growth zone, Zn metal (Sigma-Aldrich, 99.99999%, St. Louis, MO, USA) contained in an alumina boat was placed in the center of source zone of the reactor quartz tube. Initially, the reactor was kept in vacuum at a pressure of ~10^−3^ mbar. To prevent the oxidation of graphene, 500 sccm of H_2_ gas was injected while the temperature was rising at a heating rate of 20 °C/min, and the inside of the reactor was flushed until the set point was reached. When the reactor reaches the source temperature of 610 °C and the growth zone temperature reaches 700 °C, the vaporized Zn-metal was transferred into the growth zone by flowing 1000 sccm of N_2_ (99.9999%) as a carrier gas for 60 s. Here, the VPT equipment consisting of 3 source zones and 3 growth zones accurately controls the temperature of each zone with a temperature controller (K-type thermocouple, E5CB Series, RTD, PT100, OMRON, Seoul, Korea), and the substrate and Zn material were located in the center of the source and growth zone. Then, the Zn-metal layer (~20 nm) covering the graphene plays a role of a seed to grow the ZnO NR and passivates the graphene underneath. After shutting off the supply of H_2_ gas, 30 sccm of O_2_ (99.999%) gas was injected into the growth zone in the reactor, where the mixed gases of O_2_ and N_2_ flow at the rates of 1.7 and 2 standard liters per minute, respectively. The gas pressure inside the reactor was maintained at 2.4 mbar during 30 min of growth. After the reaction was complete, it was cooled to room temperature. 

### 2.3. Characterization of the Structure and Fabricated Devices 

The morphological and structural properties of the ZnO NRs grown on the graphene/Cu sheet were examined using field emission-scanning electron microscopy (FE-SEM) (XL-30, Philips, Amsterdam, Netherland) with magnification of 1.2k~12k at an acceleration voltage of 10 kV and X-ray diffraction (XRD) (SmartLab, Rigaku Corporation, Tokyo, Japan) with an X-ray source of Cu Kα radiation (λ = 1.5406 Å), respectively. The emission spectra of the temperature-dependent PL intensity were measured using Spectra Physics 404, Cryostat-HS-4, He-Cd laser-IK3202R-D, and Monochromator-SPEX 1072 in a temperature range from 10 to 140 K. The signal was amplified with analyzer-EG&G-5206. The Raman analysis was carried out using the laser excitation of 514 nm (~1 mW) polarized by a GX-AN360 (Olympus, Tokyo, Japan) filter. Each spectrum was obtained by 10 acquisitions in 10 s of accumulation time. The I-V characteristics of the glass/graphene-ZnO NRs-graphene/Cu heterostructure under dark and illumination condition of 100 μW/cm^2^ intensity (XIL-01B50KP, Newport, Irvine, CA, USA) were measured using a B1500A semi-conductor device parameter analyzer (Keysight Technologies, Santa Rosa, CA, USA) by applying a series of voltages ranging from −3 V to 3 V for dark and illumination conditions.

### 2.4. Optical Simulation

The full-wave electromagnetic simulation using Lumerical’s finite-difference time-domain (FDTD) software (FDTD, Lumerical 2020 R2.4, Vancouver, BC, Canada) was performed to analyze the optical absorption of the ZnO NRs depending on the NR distributions. The complex refractive indices (*n*, *k*) of Cu, ZnO, and graphene were taken from the References [28,29,30]. The plane wave source with a wavelength of 382 nm was injected along the z-direction (normal direction to the graphene/Cu structure). The periodic boundary conditions in both x- and y-axes and the perfectly matched layer in z-axis were implemented on the unit-cell structure. Then, the spectral absorption of the 200-nm diameter ZnO NRs on a graphene/Cu sheet were characterized by changing the inter-spacing among the ZnO NRs from 100 to 400 nm.

## 3. Results and Discussion

**Figure 1:**
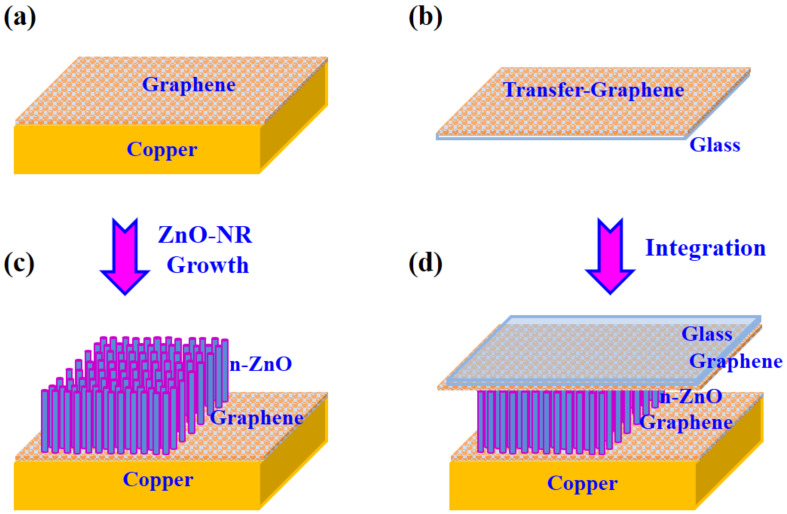
Schematic diagrams of the fabrication process for the graphene/ZnO NRs (nanorods)/graphene hybrid-structure: (**a**) Large-scale graphene prepared by the CVD (chemical-vapor-deposition) on Cu sheet; (**b**) Mono-layer graphene layer transferred to a glass substrate; (**c**) The ZnO NRs growth on the graphene/Cu sheet by the VPT (vapor-phase transport) method; (**d**) Integration of the ZnO NRs/graphene/Cu sheet with the graphene/glass substrate.

Single-layer graphene at large scale was prepared using the CVD process on Cu sheet as shown in Figure 1a [31]. The graphene-grown was cut into the size required for its purpose (here, about 1.5 cm × 1.5 cm). The graphene layer was separated from the underlying Cu sheet using Cu etchant (CE-100). The separated floating graphene layer was then transferred to a glass substrate using the fishing method (Figure 1b) [32]. Meanwhile, the graphene on the Cu sheet synthesized at 700 °C using the VPT method (Figure 1c) was utilized as the platform for the growth of ZnO NRs in such a way that the flat graphene layer on Cu sheet in vacuum condition nucleates ZnO NRs perpendicular to its surface as aforementioned. Finally, a graphene-based hetero-nanojunction device was completed by integrating the ZnO NR-graphene/Cu hybrid-structure with the graphene layer on a glass as a top electrode as shown in Figure 1d.

**Figure 2:**
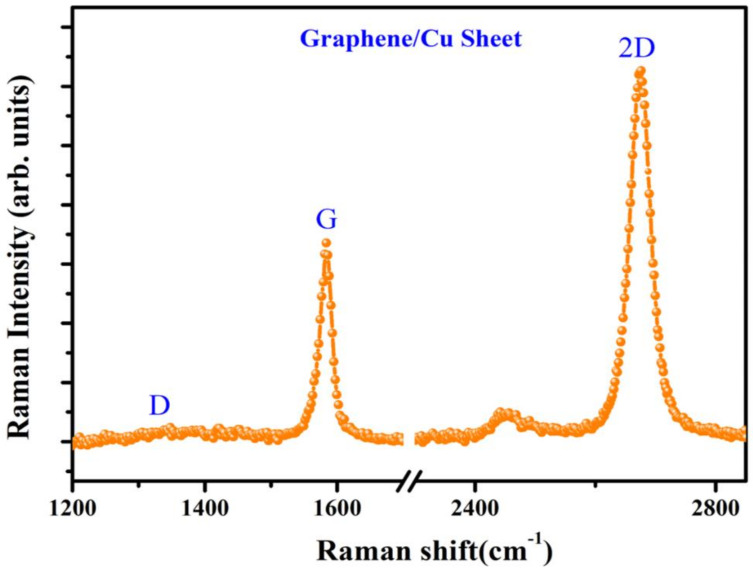
Raman spectrum of the graphene grown on Cu sheet after a background subtraction and normalization procedure.

**Figure 3:**
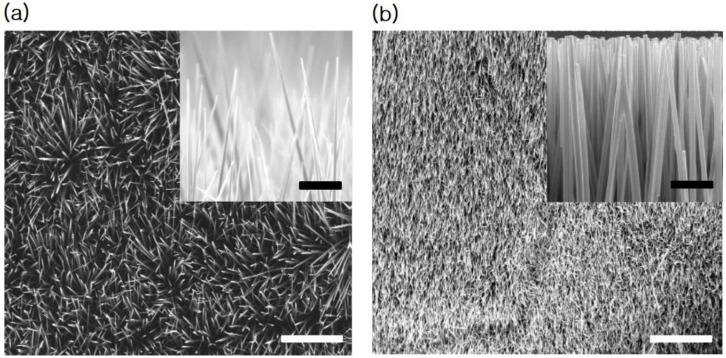
Cross-sectional view of FE-SEM (field emission-scanning electron microscopy) images (the scale bar is 20 μm) for the ZnO NRs grown on (**a**) Cu sheet and (**b**) graphene/Cu sheet using the CVD method. The inset is an enlarged view of corresponding FE-SEM images (the scale bar is 1 μm).

**Figure 4:**
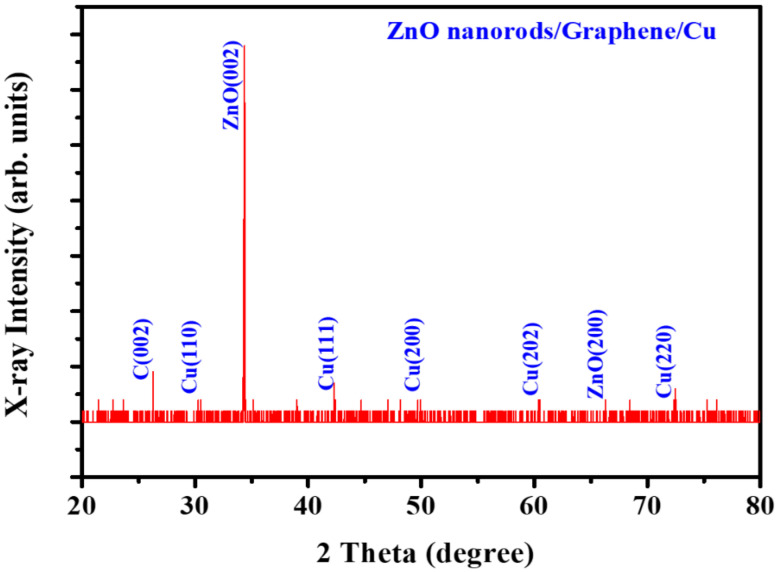
XRD (X-ray Diffraction) pattern of the ZnO NRs grown on Zn pre-deposited graphene/Cu sheet from the sample shown in Figure 3b.

To characterize the graphene on Cu sheet, Raman analysis was performed by applying a background subtraction and normalization procedure on the measured Raman spectrum since there is a high background signal due to the emission of Cu sheet. Then, it is clearly observed the main G-peak and 2D-peak of graphene at ~1584 and ~2676 cm^−1^, respectively, as shown in Figure 2. The measured spectrum reveals a typical profile of monolayer graphene with Lorentzian line shape of the 2D-peak and a peak intensity ratio of the 2D-peak to the G-peak (I_2D_/I_G_) larger than 1. Then, the morphological properties of the ZnO NRs grown on the graphene/Cu sheet in comparison with the ZnO NRs on Cu sheet were examined by cross-sectional FE-SEM images as shown in Figure 3. It is observed that the ZnO NRs on the Cu sheet (Figure 3a) are randomly oriented due to no preferential nucleation sites to grow, while the ZnO NRs (Figure 3b) are vertically well- aligned on the graphene/Cu sheet. Here, it is noted that H_2_ gas was injected to prevent the oxidation of graphene while the growth zone temperature reaches 700 °C, and then the vaporized Zn-metal was transferred to cover the graphene/Cu sheet. According to the previous research [33], the Zn droplets extracted from the reaction of ZnO/ZnOx gas with carbon layer serves as a catalyst for ZnO nanowire (NW) nucleation and growth on the surfaces of carbon materials. Thus, the pre-deposited Zn metal on graphene acts as preferential nucleation and growth sites of the ZnO NRs and protects the graphene from oxygen gas during the ZnO NR growth. Then, the fabricated ZnO NRs on graphene/Cu sheet have the length of ~20 um, a diameter of 150–200 nm, and the inter-spacing of 100–400 nm. The structural property of the ZnO NRs grown on the graphene/Cu sheet was investigated by the XRD diffraction pattern as presented in Figure 4. The XRD result shows a strong diffraction peak at 34.6° from the ZnO (002) plane or c-axis oriented, indicating a high crystallinity of the ZnO NR array as previously reported [24,34]. Here, the weak diffraction peaks coming from the carbon and Cu layer are also observed.

**Figure 5:**
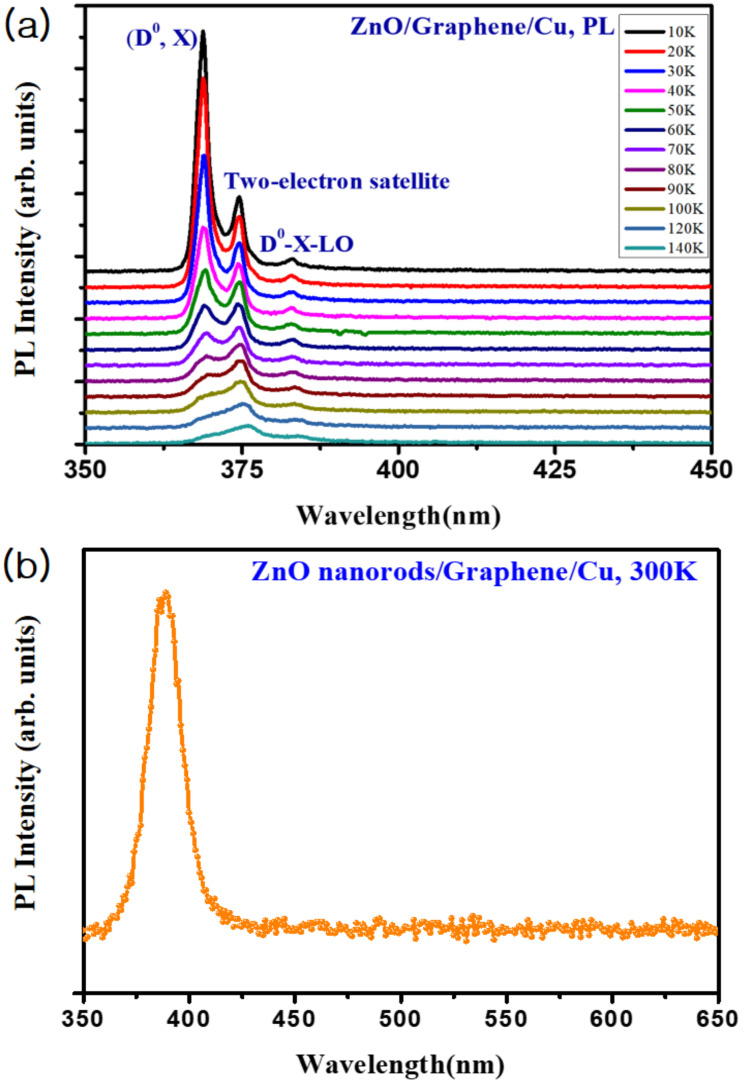
(**a**) Low temperature-dependent PL (photoluminescence) spectra in a temperature range of 10–140 K and (**b**) room-temperature PL spectrum for the ZnO NRs/graphene hybrid-nanostructure.

Low temperature-dependent PL spectra of the ZnO NRs at a temperature range from 10 to 140 K have been plotted on an arbitrary scale, as illustrated in Figure 5a. The spectrum measured at 10 K shows a dominant peak at 3.36 eV associated with the neutral-donor bound exciton. Moreover, a donor can form the excited state by the transition involving the radiative recombination from donor bound excitons. This two-electron satellite transition is attributed to the peak at 3.33 eV [35,36,37]. The peaks grouped in D°X-LO are allocated to the replicas from the neutral-donor bound excitons with a separation of 72 meV, which coincides well with the previously reported values for ZnO crystal [38]. As the temperature increases, all the peaks move towards the lower energy positions and the linewidths of the peaks are gradually broadened. This redshift of the peak position is related to a band-gap shrinkage caused by lattice increase with temperature and electron-lattice interactions [39,40]. Additionally, as the temperature increases, the gradual decrease in the intensity of the exciton emission band is attributed to breakdown of bound excitons to free excitons by higher thermionic energy [41]. The room temperature PL presented in Figure 5b show one single strong peak at a wavelength of ~382 nm, revealing no defects in the surface of the ZnO NRs. 

Generally, optical absorption in ZnO occurs only in the UV region. Since the morphology of ZnO NRs has a high surface-to-volume ratio, there can be defects on the surface of the ZnO NRs [21,22]. Then, the surface states induced by these defects lead to an absorption in a visible region [13,23]. The previous study revealed that the configuration and size of the ZnO NR play an important role in light scattering and trapping [42,43], so that the surface area related to the visible PL is ascribed from the surface states. Meanwhile, in our ZnO NRs fabricated on the graphene/Cu structure, there are no PL peaks observed in the visible wavelength except the UV region, indicating a highly crystalline ZnO NR structure. This result coincides well with the data obtained by the XRD result in Figure 4.

**Figure 6:**
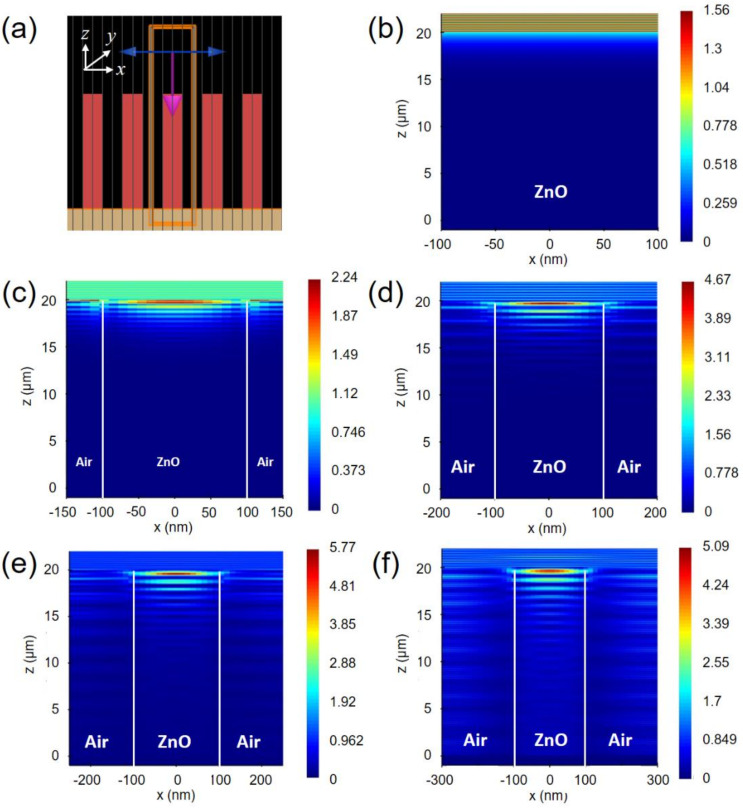
(**a**) The simulation structure for applying the FDTD (finite-difference time-domain) method. The electric-field intensity profiles inside the unit cell of the simulation structure for (**b**) 20-um thickness ZnO film and the 200-nm diameter ZnO NR arrays with an interspacing of (**c**) 100; (**d**) 200; (**e**) 300 and (**d**) 400 nm. The scale bar on the right of (**b**) to (**f**) represents the unit (V/m] of electric-field intensity.

To quantify the amount of optical absorption of the ZnO NRs depending on the NR distributions, we carried out the full-wave electromagnetic simulation using the FDTD method. The simulation structure presented in Figure 6a was based on the FE-SEM analysis (Figure 3b), considering the ZnO NR array with a diameter of 150–200 nm, inter- spacing of 100–400 nm, and a length of 20 µm. Then, the spectral absorption of the 200-nm diameter ZnO NRs on a graphene/Cu sheet was characterized by changing the inter-spacing among the ZnO NRs from 100 to 400 nm. Figure 6b shows the electric-field intensity distribution inside the 20 um-thickness ZnO layer as a reference, showing the highest intensity of 1.56 (V/m). Figure 6c–f present the electric-field intensity profiles inside the ZnO NRs with a different interspacing among the ZnO NRs of 100, 200, 300, and 400 nm, respectively. It is clearly observed that the incident light is mainly absorbed in the upper side of the ZnO NRs with the highest field intensities of 2.24, 4.67, 5.77, and 5.09 [V/m], accordingly. These results show that the optical absorption of the ZnO NRs/graphene hybrid-structure is much enhanced compared to that of thin-film ZnO counterpart (Figure 6b). This light trapping effect is highly useful to the UV light photodetection. Additionally, it is noted that the highest optical absorption occurred when the volume ratio of ZnO NRs to air is 2.5 as shown in Figure 6e. 

This result can be explained by the effective refractive index and the fraction of active region of the ZnO NR structure. The effective refractive index can be estimated by n_eff_ = n_ZnO_∙x + n_air_∙(1 − x), where n_air_ = 1, n_ZnO_ = 1.72 at a 382 nm wavelength and x is the volume fraction of the ZnO NR array with respect to the thin-film counterpart. Then, the estimated values of n_eff_ are 1.48, 1.36, 1.28 and 1.24 for the interspacing among the ZnO NRs of 100, 200, 300, and 400 nm, respectively. Hence, the ZnO NRs with the n_eff_ less than 1.3 (Figure 6e,f) lead to much reduced reflectance as compared to the ZnO NRs presented in Figure 6c,d. It is noted that the reflectance between the adjacent media is lowered as the difference in refractive indices between them is smaller. Additionally, the light absorption in the ZnO NRs is further improved since the light transmitted through the ZnO/graphene is reflected by the Cu sheet. Meanwhile, as the volume fraction of ZnO NR decreases, n_eff_ not only decreases, but also the fraction of the active region to absorb the incoming light is reduced, which means there exists a trade-off relationship between the n_eff_ and the fraction of active region of the ZnO NR array. So, the ZnO NR array presented in Figure 5e shows the highest absorption of incoming light. Therefore, the simulated results reconfirm better performance of the ZnO NR structure than a ZnO thin-film in terms of optical absorption and reveal that the amount of absorption is varied depending on the diameter and the interspacing among the ZnO NRs.

**Figure 7:**
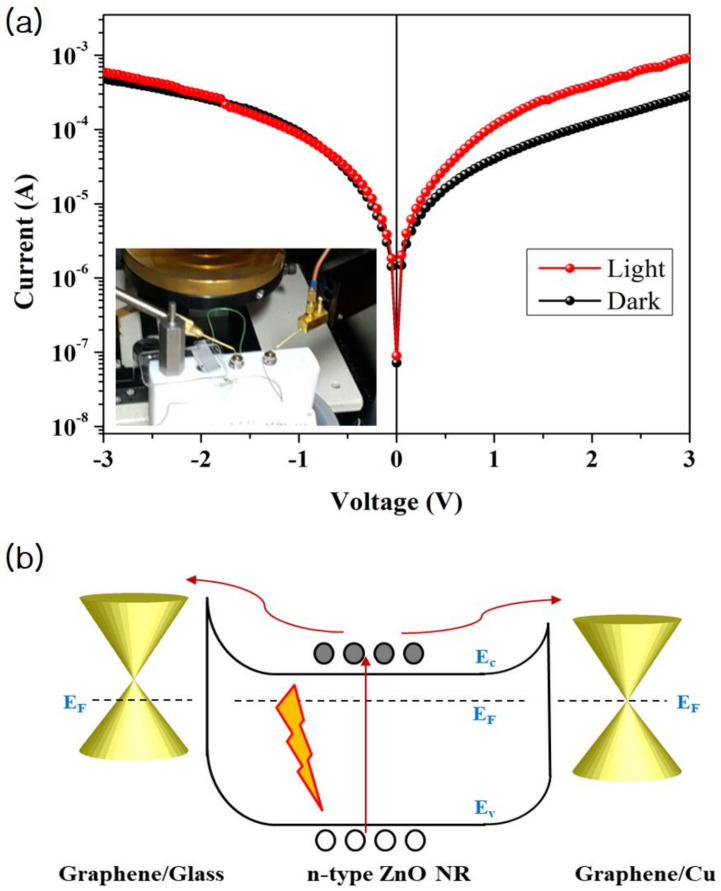
(**a**) I-V characteristics of the glass/graphene/ZnO NRs/graphene/Cu hybrid-heterostructure under dark and illuminated conditions. The inset of figure shows the photo image of an I-V measuring instrument; (**b**) Energy band diagram for the suggested nanostructure, where the white (black) circles represent holes (electrons).

To characterize the electrical properties of the graphene/n-type ZnO NR/graphene hybrid-structure, we fabricated the device in a large scale (~cm^2^) without any patterning process by simply covering the ZnO NRs/graphene/Cu sheet with the graphene/glass as a top electrode as illustrated in Figure 1. Then, the photoresponse of the fabricated heterostructures was investigated through the I-V measurement under dark and illuminated conditions. Figure 7a shows I-V characteristics in linear scale of the fabricated devices under dark and 100 μW/cm^2^ intensities of light illumination. The inset of Figure 7a shows the optical image of the I-V measuring instrument. At dark condition, the device shows asymmetrical I-V curve due to the difference in the interface of the ZnO NRs on graphene/glass and the ZnO NRs on graphene/Cu sheet. Here, the ZnO NRs were physically contacted with the graphene/glass in a large area of ~2.2 cm^2^ by a pressure, while the ZnO NRs on a graphene/Cu sheet were synthesized through the VPT method. In the ZnO NRs on the graphene/Cu sheet, the graphene has an intrinsic property because the process proceeds in a high vacuum chamber in an in situ state. Meanwhile, the electrons of the graphene in the graphene/glass structure move into the oxygen atoms contained in the glass substrate (SiO_2_) [44], resulting in p-type doping of graphene. Therefore, energy band diagram of the graphene/n-type ZnO NRs/graphene is asymmetry as shown in Figure 7b.

When the light illuminates this hybrid nanostructure, photocurrents (e.g., 1.03 mA at 3 V) arising from the ZnO NR/graphene/Cu sheet are slightly higher than that (e.g., 0.57 mA at −3 V) from ZnO NR/graphene/glass as shown in Figure 6a. The excited electrons from valence (VB) to conduction bands (CB) of the n-type ZnO NRs will transfer either the graphene/Cu sheet or the graphene/glass depending on the polarity of applied biases. When a bias voltage was applied to the top electrode of the graphene/glass, the excited electrons move towards the graphene/Cu sheet at a forward bias, while moving to the graphene/glass electrode at a reverse bias. In addition, in the ZnO NRs/graphene/Cu structure, there are almost no defects between the ZnO NRs and the graphene as shown in the XRD and PL data (Figure 4 and Figure 5). Here, the graphene layer-aided processing helps in preventing undesired defects caused by foreign metal atom catalysts and enhances ZnO nuclei nucleation [12]. Meanwhile, in the ZnO NRs physically contacted with the graphene/glass substrate, there may exist various defects in the interface during the transfer of graphene into the glass substrate, leading to a slight lower photocurrent than the case of the ZnO NRs on graphene/Cu sheet. By improving the interface states between the ZnO NRs and the graphene/glass top electrode, the photocurrent for the suggested ZnO NR/graphene-based hybrid-nanostructure can be made almost symmetrical, making a photoelectric device that can be operated in a bidirectional voltage bias.

## 4. Conclusions

The vertically aligned ZnO NRs were grown on graphene/Cu sheet using the catalyst-free VPT synthesis. The pre-deposited Zn-metal on graphene has a role of a seed to grow the highly crystalline ZnO NR without defects as well as passivating the graphene underneath. Using the graphene/Cu as a substrate also eliminates the need for a graphene transfer process, simplifying the process and avoiding physical defects of graphene caused by the transfer process. The fabricated ZnO NRs with a length of ~20 μm and a diameter of 150–200 nm shows one strong UV emission at 382 nm at room temperature. The optical absorption at UV emission of this hybrid-structure is much enhanced as compared to that of a thin-film counterpart, and the amount of absorption can be optimized according to the diameter and interspacing among the ZnO NRs. The developed graphene/ZnO NR/graphene heterojunction devices exhibit comparable photocurrents at a bidirectional voltage bias, which provides a promising way to integrate 1D and 2D nanomaterials with a cost-effective and continuous fabrication process over a large area.
